# Cancer-associated fibroblasts reveal aberrant DNA methylation across different types of cancer

**DOI:** 10.1186/s13148-024-01783-y

**Published:** 2024-11-20

**Authors:** Marco Schmidt, Tiago Maié, Thorsten Cramer, Ivan G. Costa, Wolfgang Wagner

**Affiliations:** 1 Institute for Stem Cell Biology, RWTH Aachen University Medical School, 52074 Aachen, Germany; 2 Helmholtz-Institute for Biomedical Engineering, RWTH Aachen University Medical School, 52074 Aachen, Germany; 3 Institute for Computational Genomics, Joint Research Center for Computational Biomedicine, RWTH Aachen University Medical School, 52074 Aachen, Germany; 4 Department of General, Visceral, Children and Transplantation Surgery, RWTH Aachen University Hospital, 52074 Aachen, Germany; 5 Center for Integrated Oncology, Aachen Bonn Cologne Düsseldorf (CIO ABCD), Aachen, Germany

**Keywords:** Biomarker, Cancer-associated fibroblasts, DNA methylation, Epigenetic, Human, CpG, Cancer, Hepatocellular carcinoma, Survival

## Abstract

**Background:**

Cancer-associated fibroblasts (CAFs) are essential components of the tumor microenvironment and play a critical role in cancer progression. Numerous studies have identified significant molecular differences between CAFs and normal tissue-associated fibroblasts (NAFs). In this study, we isolated CAFs and NAFs from liver tumors and conducted a comprehensive analysis of their DNA methylation profiles, integrating our finding with data from studies on other cancer types.

**Results:**

Our analysis revealed that several CAF samples exhibited aberrant DNA methylation patterns, which corresponded with altered gene expression levels. Notably, DNA methylation at liver CAF-specific CpG sites was linked to survival outcomes in liver cancer datasets. An integrative analysis using publicly available datasets from various cancer types, including lung, prostate, esophageal, and gastric cancers, uncovered common epigenetic abnormalities across these cancers. Among the consistently altered CpGs were cg09809672 (*EDARADD*), cg07134930 (*HDAC4*), and cg05935904 (intergenic). These methylation changes were associated with prognosis across multiple cancer types.

**Conclusion:**

The activation of CAFs by the tumor microenvironment seems to be associated with distinct epigenetic modifications. Remarkably, similar genomic regions tend to undergo hypomethylation in CAFs across different studies and cancer types. Our findings suggest that CAF-associated DNA methylation changes hold potential as prognostic biomarkers. However, further research and validation are necessary to develop and apply such signatures in a clinical setting.

**Supplementary Information:**

The online version contains supplementary material available at 10.1186/s13148-024-01783-y.

## Background

Cancer-associated fibroblasts (CAFs) modulate the microenvironment and are important for tumor development, metastasis, and resistance to therapy [1]. In contrast to cancer cells, CAFs usually do not harbor genetic mutations—rather, their activation is reflected in various epigenetic changes [2]. A bottleneck in the molecular characterization of CAFs is that fibroblasts generally resemble complex cell populations with striking inter- and intra-organic heterogeneity [3]. The heterogeneity of CAFs is further complicated by diverse cellular origins that can be recruited and activated during tumorigenesis, including normal-tissue-associated fibroblasts (NAFs), bone marrow-derived mesenchymal stromal cells (MSCs), stellate cells, epithelial cells, and endothelial cells [4]. This heterogeneity makes it difficult to precisely define CAFs at the molecular level and may lead to a different mechanistic contribution of CAF subtypes to cancer pathophysiology [5].

The identification of suitable molecular biomarkers for CAFs remains a major challenge [1]. Several “CAF markers”—including alpha-smooth muscle actin (αSMA), vimentin, and fibroblast activation protein alpha (FAP)—have been proposed, but their expression varies between different cancer types and CAF subpopulations [6]. To date, no single biomarker has been identified that can reliably discern CAFs and normal fibroblasts in a given tumor [7]. Integration of transcriptomic data for multiple markers into “CAF scores” has provided prognostic information for different cancer types [8], and single-cell RNA-sequencing data have revealed gene expression signatures of independent CAF subtypes that are even common to different cancer types [9]. It therefore seems conceivable that there are also epigenetic signatures of CAFs that are common to tumors of various tissues.

Changes in DNA methylation (DNAm) are tightly controlled in a very consistent manner during normal cellular differentiation. Our group and others have already shown that DNAm at single CG dinucleotides (CpGs) can provide reliable biomarkers for specific cell types, e.g., fibroblasts and MSCs [10–12]. CAFs have also been shown to have characteristic DNAm patterns for different cancer types [2, 13, 14], and it has been proposed that changes in the methylome of CAFs represent a novel epigenetic feature of the cancer microenvironment that could provide therapeutically relevant biomarkers [15]. However, so far it has been largely unclear whether such DNAm alterations are also consistent across CAFs in different cancer types [2].

In this study, we demonstrate that CAFs from liver tumors exhibit different DNAm compared to fibroblasts from adjacent tissue. A comprehensive comparison with CAF-associated DNAm in lung cancer, esophageal carcinoma, prostate carcinoma and gastric cancer demonstrated an overlap, which could potentially be indicative for the fraction of CAFs within a tumor and hence possibly be relevant for disease stratification.

## Methods

### Cell isolation and cell culture

Tissue biopsies from primary and secondary liver tumors and adjacent healthy tissues were received from the clinic for general, visceral, children and transplantation surgery at the University Hospital of RWTH Aachen after informed and written consent and following the guidelines of the ethic committee for the use of human subjects at the University of Aachen (Permit number: EK 206/09). Tissue pieces were minced into small pieces, washed with PBS and incubated at 37 °C in collagenase IV (1 mg/ml)-containing medium (KnockOut DMEM from Gibco) overnight. After filtering through a strainer, the cells were cultured in DMEM medium containing 10% human platelet lysate, Penicillin–Streptomycin (100 U/ml) and L-Glutamine (2 mM). Cells were expanded at 37 °C and 5% CO_2_ for 2–3 passages upon reaching confluence. CAFs and NAFs were successfully isolated from 11 patients (5 hepatocellular carcinoma [HCC], 5 liver metastasis of colon cancer [CRLM] and 1 liver metastasis of anal cancer [ALM]; mean age 62.6 ± 12.8 sd; 8 male and 3 female).

### Immunostaining

For the immunostaining, cells were cultured on gelatin (0.1%)-coated cover slips and fixed with 2–4% PFA for 15 min. Cells were permeabilized for 30–60 min with 0.5% TWEEN 20 or 0.1% Triton™ X in PBS containing 5% BSA. Primary antibodies for vimentin (Sigma-Aldrich), α-SMA (Sigma-Aldrich) and pan-cytokeratin (Sigma-Aldrich) were added overnight at 4 °C (Supplemental Table S1). The next-day secondary antibodies Alexa 594 and Alexa 647 (ThermoFisher) were added for 1 h. Nuclei were counter-stained with DAPI.

### Flow cytometry

Cells were fixed in 2% PFA for 15 min and stained with conjugated antibodies for 30 min. These included mouse-anti-human antibodies for CD14, CD29, CD31, CD34, CD45, CD73, CD90, and CD105 (Supplemental Table S1). Afterward, cells were kept in PBS with 2% FCS. Samples were measured with a FACSCanto II (BD Biosciences), and the FlowJo software was used to analyze the data.

### DNA methylation analysis

Genomic DNA was isolated with the NucleoSpin Tissue kit (Macherey-Nagel) and hybridized to Illumina MethylationEPIC BeadChips (at Life and Brain, Bonn, Germany). Initial quality control of DNA methylation data was performed with the minfi package (v1.48.0), and three samples with low overall signal intensities were removed at this step. The SeSAMe package (v1.20.0) [16] was used for preprocessing (“QCDPBG”) including dye bias correction, quality mask filtering [17], NOOB normalization [18] and calculation of detection *p*-values. CpG probes, which failed in 10% or more samples, non-cg probes, probes on X- and Y-chromosomes or probes flagged in the b5 manifest were removed. The data were then converted into a GenomicRatioSet to apply minfi-based functions. Additionally, we removed two outlier NAF samples.

For the public data from GEO (Supplemental Table S2) .idat files were preprocessed in the same way. The data were preprocessed separately as three different datasets and then merged. If no .idat files were available, we utilized signal intensities and beta matrices. The public data consisted of Illumina 450 K and EPIC data and were therefore reduced to the probes that overlap.

We used the Limma (v.3.58.0) package to generate PCA plots with the plotMDS function (gene.selection = "common"), and the differential methylation analysis, where probes with ≥ 0.2 difference in mean beta values and adjusted *p*-values (Benjamini-Hochberg) ≤ 0.05 were considered significant. The ComplexHeatmap package (v2.18.0) was used to generate the heatmaps including Pearson correlation as distance and the “ward.D2” method for clustering. Gene ontology analysis was done with the missMethyl R package (v.1.36.0). The DMRcate package (v2.14.1) was used to identify differently methylated regions, which were defined as regions of 1000 bp that included at least 2 differentially methylated CpGs with a “betacutoff” of 0.2 and a “pcutoff” of FDR = 0.05. The UpSetR package (v1.4.0) was used to make the upset plots. The DMR methylation plots were done using the Gviz (v1.46.0) and org.Hs.eg.db R packages (v3.18.0). To calculate the epigenetic age of the samples, the wateRmelon R package (v2.8.0) was used.

For selection of top ranked CAF-specific CpGs we used our previously developed R package CimpleG (v0.0.5.9001) [19]. For this purpose, we divided the samples randomly into a selection and test set based on an 80/20 split. We also performed a pre-selection of CpGs based on at least 20% mean difference in methylation values between all NAFs and CAFs.

### RNA-seq analysis

Total RNA was isolated using the NucleoSpin RNA kit (Macherey–Nagel). For library preparation, the TruSeq-Stranded mRNA kit (Illumina) was selected and sequenced on a NextSeq 500 (Illumina) using the NextSeq 500/550 High Output Kit v2.5 (150 cycles). Sequencing was performed at IZKF-associated Genomics Facility of the RWTH University. The nf-core/rnaseq pipeline was applied for alignment using STAR (hg38 genome) and generation of the count matrix using Salmon. Analysis was done with DESeq2 in R [20]. Generally, genes with overall less than 10 counts were removed. Genes were considered significantly differentially expressed when they showed a log2 fold change ≥ 2 and adjusted *p*-value ≤ 0.1. For visualization , the data were VST transformed. RNA-seq and DNA methylation data were matched and combined by annotating both to Ensemble gene IDs.

### Survival analysis

Data gathering, curation and analysis was performed in R. Data for all TCGA projects were downloaded with the TCGAbiolinks R package (v2.28.3), setting the parameters data.category to “DNA Methylation,” sample.type to "Primary Tumor," platform to "Illumina Human Methylation 450" and data.type to "Methylation Beta Value." Any sample that did not have information for the CpGs under analysis was dropped. Focusing on the clinical data, samples that showed up as duplicated or for which the survival clinical variables (deceased, days_to_death and days_to_last_follow_up) could not be found were not considered. For the Kaplan–Meier plots and log-rank tests, cancer patients were stratified by the 25th percentile of DNAm at the respective CpG. For the Cox proportional hazards models, the continuous methylation value was used for each CpG (cg09809672, cg07134930, cg05935904) with gender and age as additional model variables. The *p*-value associated to each model variable was corrected using Benjamini and Hochberg *p*-value correction procedure.

## Results

### Aberrant DNA methylation in cancer-associated fibroblasts from liver tumors

**Fig. 1:**
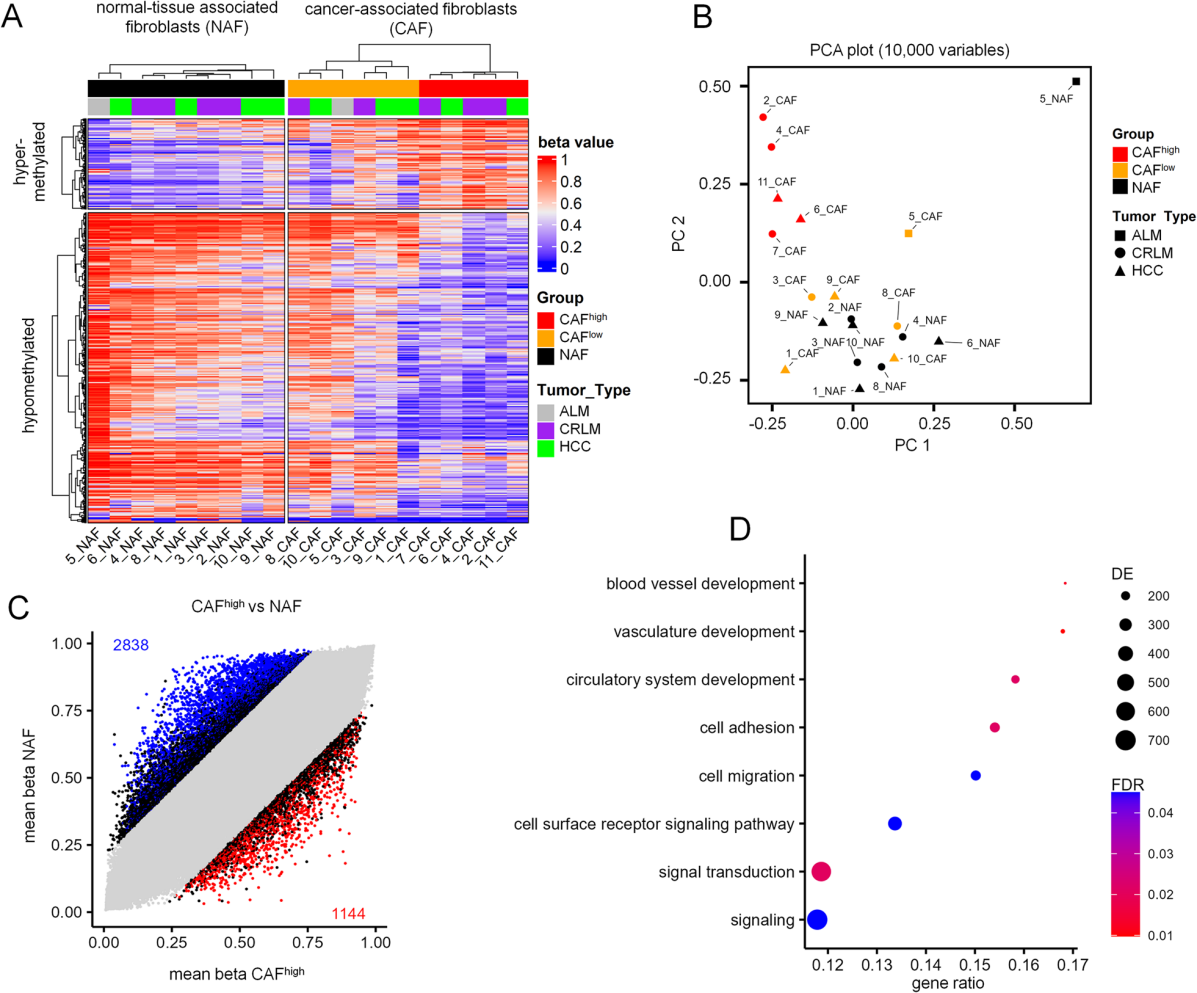
Aberrant DNA methylation in fibroblasts of liver cancer. **A** Liver fibroblasts were isolated from cancer tissue (CAFs) or from tumor-free neighboring tissue (NAFs) and analyzed on EPIC bead chips. The heatmap depicts DNA methylation at 2,134 CpGs with at least 20% mean methylation difference between NAFs and CAFs. Hierarchical clustering showed two groups of CAFs, which were referred to as CAF^low^ (orange) and CAF^high^ (red). **B** The principal component analysis (PCA) of the 10,000 most variable CpGs showed that CAF^high^ clustered apart from CAF^low^ and NAFs. **C** Scatterplot comparing the mean beta values of the CAF^high^ group *versus* NAFs. Significant differentially methylated CpGs are highlighted (mean DNAm difference > 20%; limma adjusted *p*-values < 0.05). **D** Gene ontology enrichment („biological process”) of CpG sites with significant differential DNAm between CAF^high^ and NAFs (DE = number of differentially methylated genes, FDR = false discovery rate)

Fibroblasts were isolated from cancer (CAFs) and from tumor-free adjacent tissue (NAFs) of hepatocellular carcinoma (HCC) or liver metastasis of colorectal/anal cancer (in total n = 11 CAF/NAF pairs). All cell preparations showed typical fibroblastoid morphology and surface marker expression (CD14^−^, CD29^+^, CD31^−^, CD34^−^, CD45^−^, CD73^+^, CD90^+^, and CD105^+^). In addition, immunostaining demonstrated that they were positive for vimentin, negative for pan-cytokeratin, and heterogeneous for alpha-smooth muscle actin (α-SMA), indicating that all cell preparations could be classified as fibroblastoid cells (Supplemental Figure S1). The DNA methylation profiles were then analyzed with Illumina MethylationEPIC BeadChips (two outlier NAF profiles were removed). For orientation, we initially selected 2,134 CpG sites with at least 20% difference in mean DNA methylation in CAFs *versus* NAFs. Hierarchical clustering of these CpGs indicated that the CAF profiles can be categorized in two groups, with one group being closer related to NAFs. Since we anticipated heterogeneity within CAFs—particularly since we did not use surface markers to enrich specific subfractions—we subsequently refer to these two clusters as CAF^high^ and CAF^low^ (Fig. 1A). The CAF^high^ samples were also clearly separated in a principal component analysis (PCA) plot of the 10,000 most variable CpG sites (Fig. 1B). This suggests that CAF-specific epigenetic aberrations vary between samples, and we therefore focused particularly on the epigenetic differences between CAF^high^ and NAFs.

Differential methylation analysis (> 20% difference in mean methylation; limma adjusted *p*-values < 0.05) revealed 2,838 significantly hypomethylated and 1,144 hypermethylated CpGs (Fig. 1C; Supplemental Figure S2A,B; Supplemental Table S3). Gene ontology analysis revealed that aberrant DNA methylation was significantly enriched in categories associated with angiogenesis, cell migration, and signaling (Fig. 1D). The differently methylated sites appeared to be located in gene bodies rather than promoter regions (Supplemental Figure S2C). The most significantly hypomethylated region was in the collagen type I alpha 1 chain (*COL1A1*) gene, a gene that has been used in the literature as a gene expression marker for a subset of CAFs [8, 9, 21], and the top hypermethylated region was in the gap junction protein alpha 4 (*GJA4*; Supplemental Figure S2D,E). These differentially methylated regions might reflect the activation of CAFs in liver tumors.

### *Cancer*-associated fibroblast reveal aberrant gene expression that reflects epigenetic aberrations

**Fig. 2:**
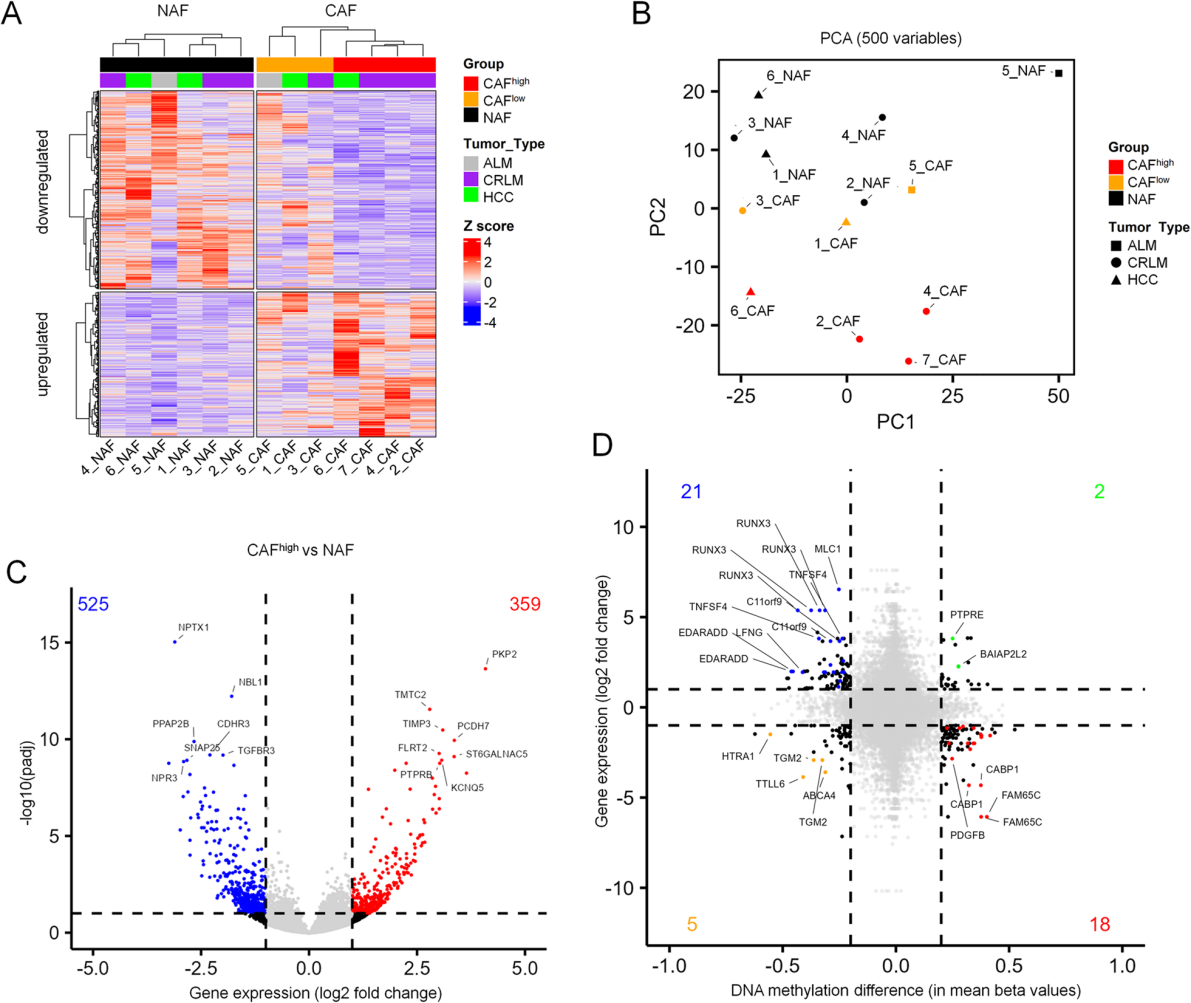
Gene expression differences between CAFs and NAFs from liver. **A** Heatmap of 891 genes with at least fourfold expression difference between the groups of NAFs and CAFs. Hierarchical clustering showed the same classification of CAF^low^ (orange) and CAF^high^ (red), as observed for DNAm. **B** Principal component analysis of the 500 most variable genes. **C** Volcano plot comparing the CAF^high^ group with NAFs. Highlighted are significantly different expressed genes (adjusted *p*-values < 0.1). **D** Differential gene expression was compared with differential methylation in CAF^high^ *versus* NAFs. Each CpG site in promoter regions (TSS1500 and TSS200) was paired with the associated genes. Highlighted are significantly differentially expressed and methylated genes

To better understand if the aberrant DNAm in CAFs is also reflected at the gene expression level, we performed RNA-sequencing on seven of the NAF/CAF pairs. Analogous to the DNA methylation analysis, we initially selected at least fourfold difference in gene expression between NAFs and CAFs. This analysis resulted in the same separation of CAF^high^ and CAF^low^ samples as previously observed (Fig. 2A,B). Further analysis of differential gene expression between the CAF^high^ and NAF group revealed 359 upregulated and 525 downregulated genes (Fig. 2C; Supplemental Figure S3). Among the most significant upregulated genes were plakophilin-2 (*PKP2*), which has been described as a Wnt/β-catenin target in colon cancer CAFs [22], and TIMP metallopeptidase inhibitor 3 (*TIMP3*), which was also highly upregulated in ovarian cancer CAFs [23].

We then analyzed whether the aberrant DNA methylation in the CAF^high^ fraction was also reflected in corresponding changes in gene expression. In general, hypomethylation was rather associated with higher gene expression, and the opposite was also true—but the association between differential DNAm and corresponding gene expression changes was overall low (Fig. 2D). Interestingly, *EDARADD* was among the genes that showed high expression and hypomethylation. This gene has previously been independently described as differentially methylated and expressed in CAFs from lung and prostate cancer [13, 24]. In addition, the Runt-related transcription factor 3 (*RUNX3*) was among the highest expressed genes, as previously described for tumor-supporting CAFs from breast cancer [25].

### Selection of candidate CpGs for *cancer*-associated fibroblasts in liver tumors

**Fig. 3:**
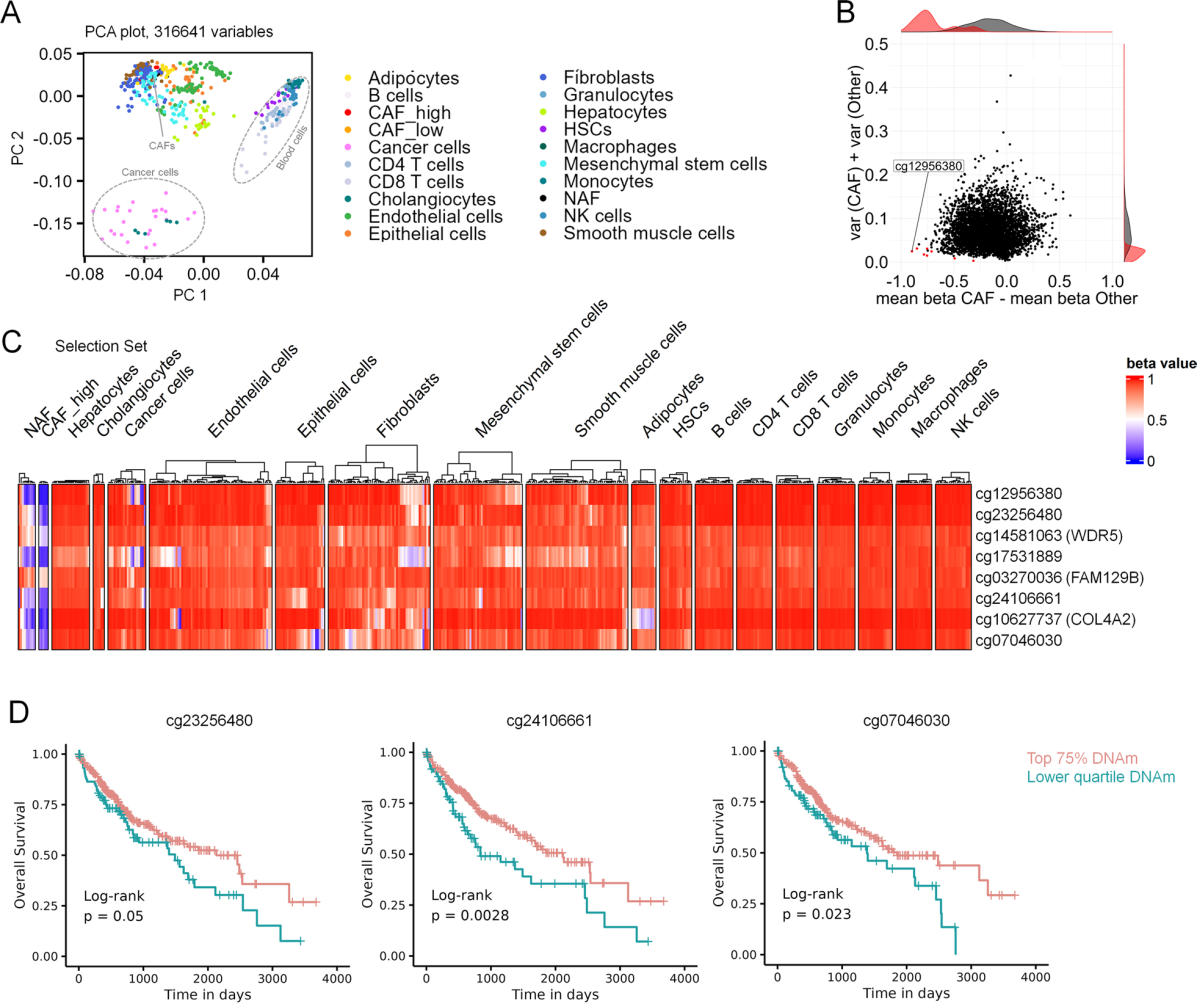
Selection of potential DNA methylation biomarkers for CAFs in liver cancer. **A** Principal component analysis of DNA methylation profiles (316,641 CpGs) in NAFs, CAFs with public datasets of various other cell types. Our NAFs and CAFs clustered closely to fibroblasts of other studies. **B** The selection of candidate CpGs was performed with CimpleG [19] on a reduced number of CpGs, that showed at least 20% mean methylation difference between NAFs and CAFs. **C** Heatmap of DNAm of eight candidate CpGs that were selected to discern CAFs from other cell types. The results of the selection dataset are depicted here. **D** To investigate if DNA methylation at these eight candidate CpGs is associated with overall survival, we used the TCGA data of hepatocellular carcinoma [38]. Kaplan–Meier analysis of the 25th percentile of patients with the lowest DNA methylation at these sites *versus* other patients revealed significant results for three CpGs (cg24106661; cg07046030; and cg23256480)

We hypothesized that an epigenetic biomarker for CAFs might reflect the fraction of CAFs in liver tissue, which might be useful for disease stratification of liver cancer. However, since liver tissue comprises many other cell types other than fibroblasts (such as hepatocytes, endothelial cells, blood cells etc.), their profiles need to be taken into consideration. We therefore searched for CpG sites with distinct DNAm levels between CAFs and other cell types that are present in liver tissue, including liver cancer cells. To this end, we compiled a dataset of in-house and public methylation data with a wide range of different cell types that might be found in liver, albeit they were not sorted from liver tissue (Supplemental Table S2). CAFs, NAFs and fibroblasts were close to each other in the PCA plot, while hematopoietic cells and cancer cell lines/cholangiocyte cancer cells were separated into distinct clusters (Fig. 3A). Notably, methylome of CAFs was closely related to that of fibroblasts, supporting the notion that CAFs are of fibroblastoid origin.

To identify liver CAF-associated CpGs, we divided the datasets into a selection set (including CAF^high^ samples) and test set (with CAF^low^ samples). We used CimpleG with a preselected number of CpGs (> 20% mean difference between NAFs and CAFs) to select candidate CpGs (Fig. 3B) [19]. We exemplified the top eight candidate CpGs, all of which were hypomethylated in the CAF^high^ samples and consistently methylated in other cell types in the selection set (Fig. 3C) as well as in the test set (Supplemental Figure S4A). We then tested whether the eight candidate CpGs for CAFs in liver tissue would also reveal different DNAm in hepatocellular cancer compared to normal liver tissue of another study [26]. In fact, DNAm was overall lower in cancer samples, but this was not consistent for all CpGs and cancer samples (Supplemental Figure S4B). Furthermore, we analyzed liver hepatocellular carcinoma profiles of The Cancer Genome Atlas (TCGA) and the CAF-candidate CpGs revealed higher variability in cancer tissue (Supplemental Figure S4C).

To estimate whether the DNAm pattern in these CAF-associated CpGs is also indicative for prognosis in liver cancer, we compared the 25th percentile of patients with the lowest methylation at these sites with other patients. Three of the eight CpGs showed a clear association with survival (cg24106661; cg07046030; and cg23256480; Fig. 3D). Thus, lower DNA methylation at these CpGs may reflect a higher proportion of CAFs contributing to shorter long-term survival in hepatocellular carcinoma.

### Aberrant DNA methylation of *cancer*-associated fibroblasts in different types of *cancer*

**Fig. 4:**
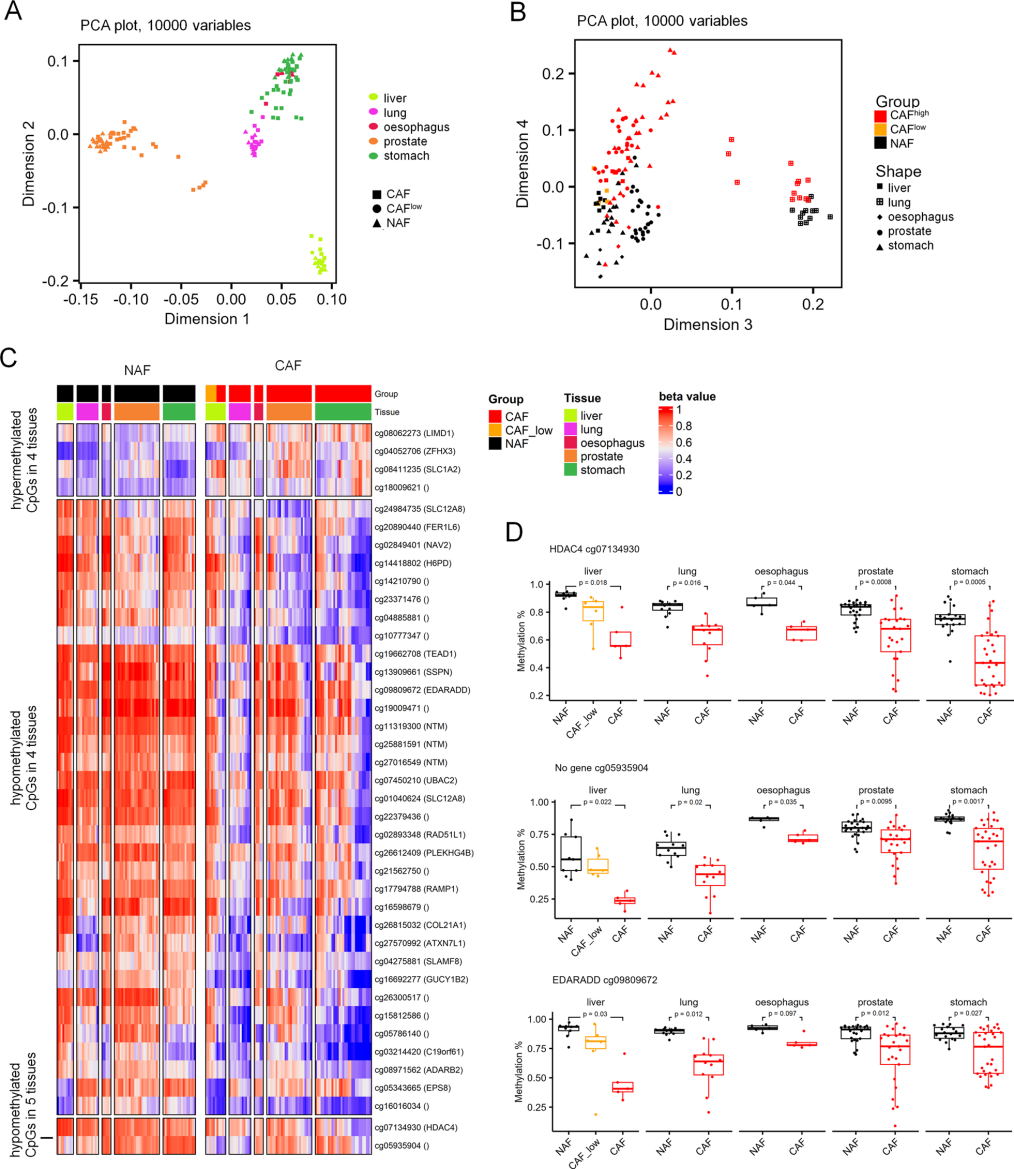
Aberrant DNA methylation in fibroblasts associated with various types of cancer. **A**, **B** Principal component analysis of the 10,000 most variable CpGs in the dataset containing NAFs and CAFs from lung (GSE68851) [24], esophagus (GSE97687)[27], prostate (GSE115413 and GSE86258) [13], and stomach cancer (GSE117087, GSE194259 and GSE97686) [27, 28]. The samples clustered primarily according to the tissue in dimensions 1 and 2 (**A**), whereas they were separated into NAFs and CAFs by the fourth dimension (**B**). **C** Heatmap of 36 hypomethylated and 4 hypermethylated sites in CAFs versus NAFs, which were significantly differentially methylated in at least 4 of the tissues/cancers (mean methylation difference between NAFs and CAFs > 10%; limma adjusted *p*-values < 0.05). **D** Box plots of DNA methylation levels for all samples for the four differently methylated hypomethylated sites shared by all five tissues (adjusted *p* values are based on the limma differential methylation analysis)

Subsequently, we have analyzed if our liver CAF-associated CpGs are overlapping with CAF-associated CpGs in other types of cancer. To this end, we used publicly available Illumina BeadChip profiles of CAFs and NAFs: from non-small cell lung cancer [24], prostate cancer [13], adenocarcinomas of the stomach and esophagus [27], and three other datasets with CAFs from gastric cancer [27, 28] (Supplemental Table S2). The samples were mainly grouped according to the tissue of origin in dimensions 1 and 2 in the PCA plot (Fig. 4A). Notably, component 4 of the PCA analysis separated CAF from NAF samples across all five different tissues, indicating that there may indeed be overlapping epigenetic differences (Fig. 4B).

Subsequently, we performed differential methylation analysis between NAFs and CAFs for each cancer type separately. CpGs were selected where the difference in mean DNAm > 10% and the limma adjusted *p*-values was < 0.05. Significant hypo- and hypermethylation was found for 1,134 and 559 CpGs in liver, 923 and 596 CpGs in lung, 9,631 and 5,199 CpGs in prostate, 1,389 and 210 CpGs in esophagus, and 3,286 and 2,988 CpGs in stomach, respectively. Of note, 34 hypomethylated and four hypermethylated sites were shared by at least four of these cancer categories (Fig. 4C, Supplemental Figure S5A,B). Furthermore, two hypomethylated CpGs were found in common in all datasets: cg07134930 in histondeacetylase 4 (*HDAC4*), and cg05935904 (not related to a gene; Fig. 4D).

As previously mentioned, *EDARADD* has been described as one of the most differentially methylated regions in CAFs in two independent studies of lung and prostate cancer [13, 24]. Notably, the same CpG site cg09809672 (*EDARADD*) was significantly differently methylated in all our comparisons except for esophagus. Moreover, this CpG site has also been described as one of the genomic sites with conspicuous age-associated DNA methylation changes [29]. However, when we used three different epigenetic clocks on CAFs and NAFs from liver [30–32], there were no significant differences in epigenetic-age predictions (Supplemental Figures S5C), which was in line with previous reports for prostate CAFs [13]. Overall, our integrative analysis indicated that CAFs from different tissues have overlapping epigenetic features.

### DNA methylation at CAF-associated CpGs is indicative for prognosis

**Fig. 5:**
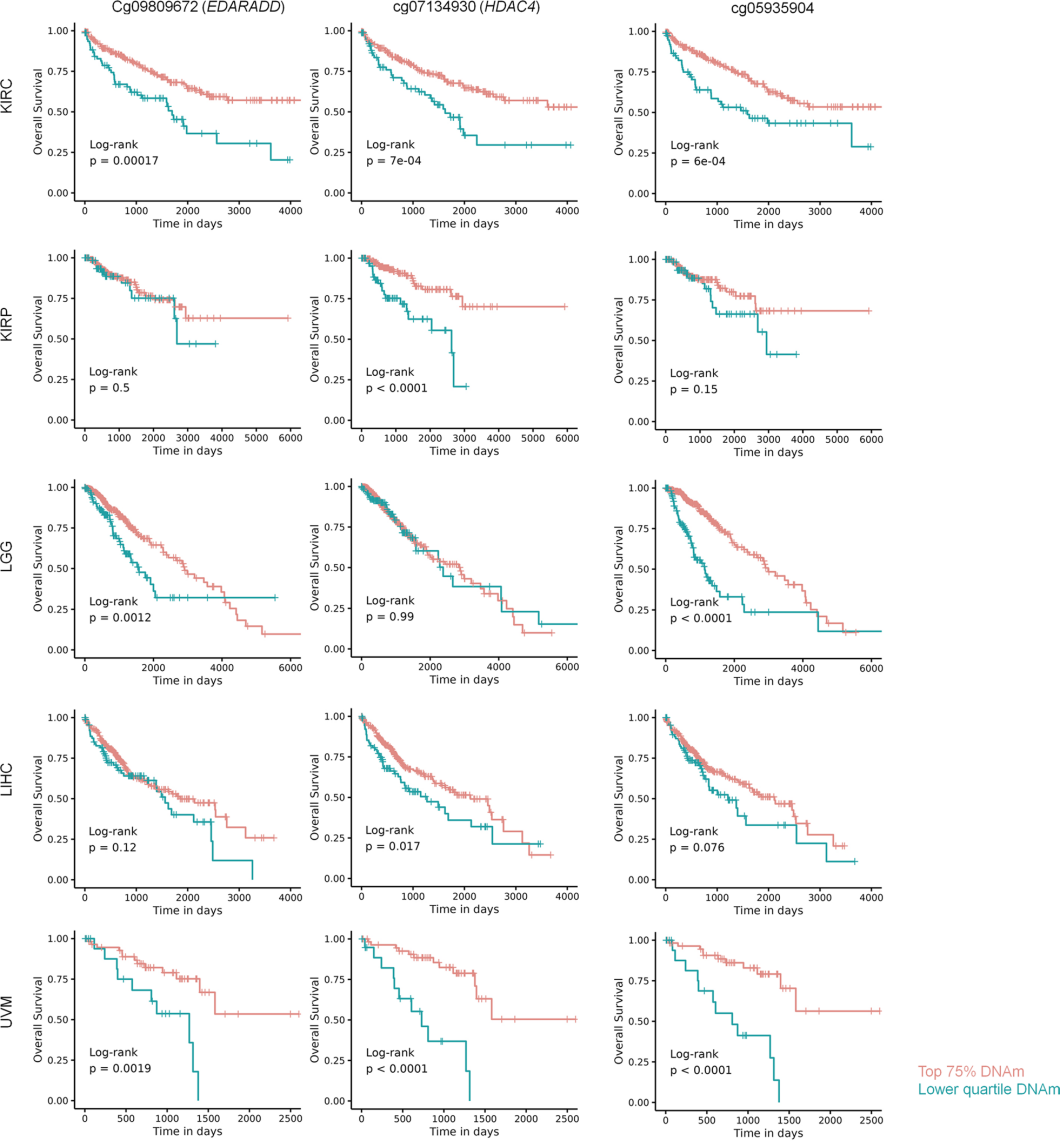
DNA methylation of cancer-associated fibroblasts is indicative for overall survival. Kaplan–Meier plots with overall survival for TCGA DNA methylation data of five different cancers: kidney renal clear cell carcinoma (KIRC), kidney renal papillary cell carcinoma (KIRP), low grade glioma (LGG), liver hepatocellular carcinoma (LIHC), and uveal melanoma (UVM). Patients were stratified by the 25th percentile of lowest DNA methylation at the three CAF-associated CpGs (cg09809672 in *EDARADD*, cg07134930 in *HDAC4*, and cg05935904 without gene-association). Hypomethylation at these CpGs seems to be associated with higher CAF-content and shorter overall-survival

The prevalence of cancer-associated fibroblasts might be associated with adverse prognosis in various types of cancer [1]. We have therefore investigated if the DNA methylation levels in the CAF-associated CpGs cg09809672 (*EDARADD*), cg07134930 (*HDAC4*), and cg05935904 were indicative for overall survival. Our analysis encompassed 32 datasets from diverse cancer types within The Cancer Genome Atlas project (TCGA). To estimate association with overall survival we initially performed Cox proportional hazards models, incorporating age and gender as variables. In five cancer types—liver hepatocellular carcinoma (LIHC), kidney renal clear cell carcinoma (KIRC), kidney renal papillary cell carcinoma (KIRP), low grade glioma (LGG), and uveal melanoma (UVM)—at least one of the three CpGs revealed a significant association with survival (Supplemental Table S4). Furthermore, the association between the DNAm profiles of the three CpGs with survival was supported by Kaplan–Meier curves and log-rank test, comparing the 25th percentile of patients with the lowest methylation at these sites with the remaining patients (Fig. 5).

## Discussion

Our results provide further evidence that interaction of cancer cells with their environment systematically remodels the epigenetic makeup of CAFs. Despite the heterogeneity of CAFs within a given tumor, among diverse tissues, and across individuals, there were consistent DNA methylation changes observed in multiple independent datasets. While not all liver-CAF samples exhibited distinct signatures, it is conceivable that the CAF^low^ samples encompass either less activated fibroblasts or distinct subpopulations. Previous research has identified different CAF-clusters [8, 33] and it was even speculated that CAF subpopulations expressing different biomarkers might either promote or counteract tumor growth [4, 7, 34]. In addition, Ma et al. have recently delved into the heterogeneity of CAF subsets using single-cell gene expression data across different cancer types [35]. It is conceivable that such diversity in CAF-subsets may also manifest in their methylome at the single-cell level, warranting further investigation in future studies. Either way, the CAF^low^ fraction revealed similar DNAm and transcriptomic patterns as NAFs. Furthermore, analysis of methylome and transcriptome data consistently identified CAF^high^ samples with abnormal expression, particularly in candidate CAF-markers such as *RUNX3* [25] and *EDARADD* [13]. The heterogeneity in CAFs in transcriptome and DNAm might also originate from different cellular sources recruited into the CAF compartment [4]. Nevertheless, our analysis suggests that CAF-associated DNAm profiles closely resemble those of normal fibroblasts, hinting at a fibroblastoid cellular origin.

Reliable biomarker discovery for CAFs may eventually assist patient stratification and ultimately provide new targets for therapeutic approaches [34]. In this study, we employed our CimpleG pipeline as a proof of concept to identify candidate CpGs for CAFs in liver tumors. Notably, hypomethylation at these CpGs was particularly observed in CAFs, to a lesser degree in NAFs, and exhibited high methylation levels in all other cell types examined. While our study establishes an association between hypomethylation at three of the eight tested CpGs and shorter overall survival in liver cancer, it is important to note that these biomarkers were selected based on a relatively small set of CAF samples. Consequently, further validation across independent cohorts is warranted in future studies.

It was remarkable to observe that apparently similar genomic regions become hypomethylated in CAFs across various studies and cancer types. Specifically, the three tested CpGs (cg09809672 in *EDARADD*, cg07134930 in *HDAC4*, and cg05935904) exhibited very consistent hypomethylation in CAFs compared to NAFs across all studies examined in our cross-comparison. In addition, Su and coauthors investigated DNAm differences between CAFs and NAFs of non-small cell lung cancer (NSCLC) and published a table with 14,781 differentially methylated CpG sites [36]—notably, their selection also comprised all of our three above-mentioned CpG sites. This overlap further substantiates our finding that these CpGs have aberrant DNAm in CAFs of different types of cancer.

In a prior study, Zou and coworkers focused on gene expression data to identify a signature of seven genes that were highly expressed in fibroblasts, and upregulated in ovarian cancer stroma compared with normal ovarian stroma [8]. Notably, their candidate genes comprised *COL1A1*, which featured one of the most prominent hypomethylated regions in our CAF^high^ fractions, and podoplanin (*PDNP*), showing differential methylation in CAF^high^ *versus* NAFs. Their research linked elevated expression of this CAF signature with unfavorable prognosis in various cancer types. Our findings align with this association, revealing a similar link between hypomethylation at the three identified CpGs in CAFs and adverse outcomes across up to five different cancer types, with particularly pronounced effect in kidney renal clear cell carcinoma (KIRC). Importantly, our analysis does neither prove that the CAF-associated DNAm changes are functionally relevant, nor that these patterns directly affect clinical outcome—particularly given that there is huge variation between patients and tumors.

Our findings support the notion that the tumor microenvironment differs in those samples that have higher or lower CAF-associated DNAm patterns. Unfortunately, the fraction of CAFs within the tumor samples of the available datasets is unknown, and hence, we can only speculate that CAF-associated DNAm might reflect differences in the tumor microenvironment. It is conceivable that CAF-associated DNAm changes might ultimately be considered as a supportive biomarker in the future, but this would probably require an application-specific derivation of such signatures and certainly further validation.

*Our study has several limitations* The sample size of the analyzed liver tumors is very limited. Furthermore, CAFs are notoriously heterogeneous and tumors will also comprise fibroblasts that are not activated by the cancer microenvironment. We did not use specific CAF markers to enrich for activated subsets [37]—and it is yet unclear if such markers can be used for enrichment form liver cancer at all. Furthermore, there remain many questions on the clinical and functional relevance on the epigenetic CAF signatures. While there was an overlap in aberrant DNAm in CAFs from various tissues, it is still unclear if the signatures can really reflect the fraction of CAFs within a tissue specimen. Thus, it remains to be proven that the association with survival in the different cancer samples is really attributed to the fraction of activated CAFs. The relevance for clinical prognosis needs to be further validated in independent datasets and studies.

## Conclusions

Our exploratory study highlights that CAFs from liver cancer have distinct DNAm patterns to NAFs. Moreover, these DNAm patterns exhibit notable overlap with CAF-signatures identified in other studies across various cancer types. This consistency suggests that there are reproducible epigenetic modifications occurring during the activation of CAFs. In the future, it needs to be further analyzed if epigenetic signatures can reliably capture the fraction of activated fibroblasts in cancer tissue, which might eventually even be considered for therapeutic decisions.

## Supplementary Information

Additional file1 (PDF 1603 KB)

Additional file2 (XLSX 53 KB)

Additional file3 (XLSX 435 KB)

## Declarations

### Availability of data and materials

DNA methylation profiles generated in this study can be accessed from the Gene expression omnibus (GEO) under accession number GSE255123; the RNA-sequencing data are accessible under GSE255122. The following tokens have been created to allow review of the records GSE255123 (qxahsaionterlqr) and GSE255122 (khmjmegabnmjnqn).
