# Supplementary material for: Cancer-associated fibroblasts reveal aberrant DNA methylation across different types of cancer
Source: Clin Epigenetics. 2024 Nov 20;16:164. doi: 10.1186/s13148-024-01783-y (PMC11580436; doi:10.1186/s13148-024-01783-y)
Supplement: Supplementary file 1 — Additional file1 (PDF 1603 KB) [file 13148_2024_1783_MOESM1_ESM.pdf]

Supplemental Data

Cancer-associated fibroblasts reveal aberrant DNA methylation across different types of cancer

Marco Schmidt[1,2], Tiago Maié[3], Thorsten Cramer[4], Ivan G. Costa[3], Wolfgang Wagner[1,2,5,*]

1 Institute for Stem Cell Biology, RWTH Aachen University Medical School, 52074 Aachen, Germany
2 Helmholtz-Institute for Biomedical Engineering, RWTH Aachen University Medical School, 52074 Aachen, Germany
3 Institute for Computational Genomics, Joint Research Center for Computational Biomedicine, RWTH Aachen University Medical School, 52074 Aachen, Germany
4 Department of General, Visceral, Children, and Transplantation Surgery, RWTH Aachen University Hospital, 52074 Aachen, Germany
5 Center for Integrated Oncology Aachen Bonn Cologne Düsseldorf (CIO ABCD), Aachen, Germany
* Correspondence: wwagner@ukaachen.de

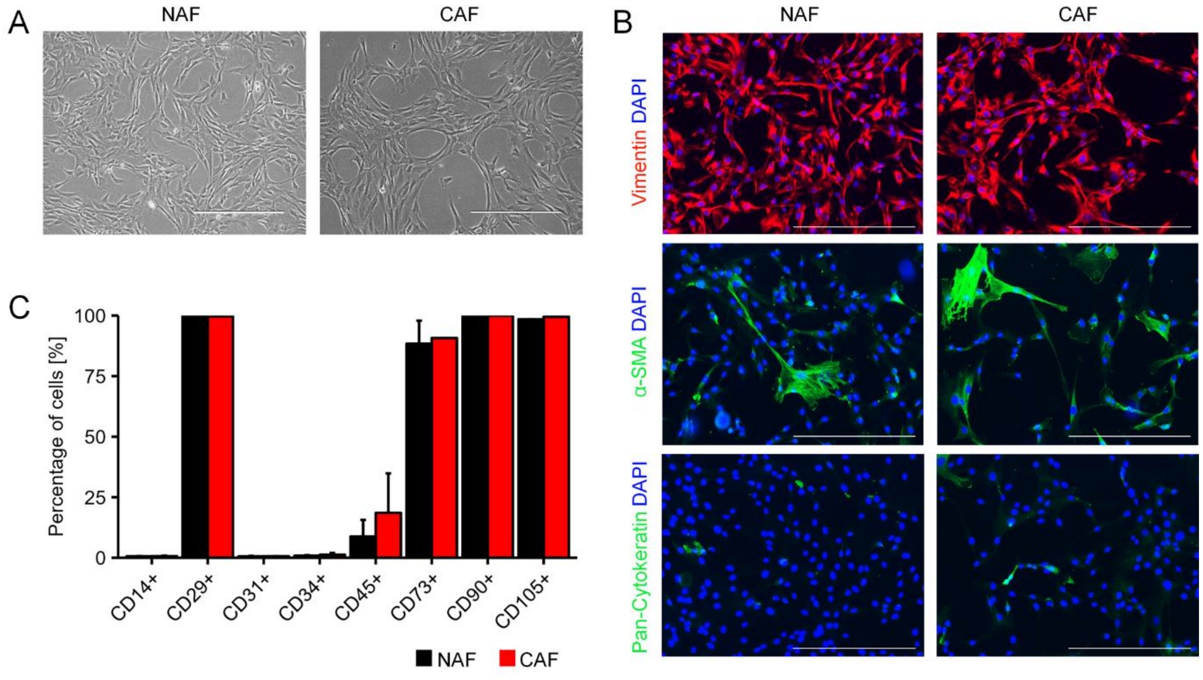

Figure S1: Characterization of CAFs from liver.

(A) Phase contrast images of normal tissue-associated fibroblasts (NAFs) and cancer-associated fibroblasts (CAFs) from one exemplary donor. Scale bar = 400 µm.

(B) Immunostaining of NAFs and CAFs from one exemplary donor. Stained with antibodies for vimentin, alpha smooth muscle actin (αSMA), and pan-cytokeratin. Scale bar = 400 µm.

(C) Flow cytometry results across different donors (n = 9). All cell preparations represent a typical fibroblastoid immunophenotype and are positive for CD105, CD29, CD73 and CD90.

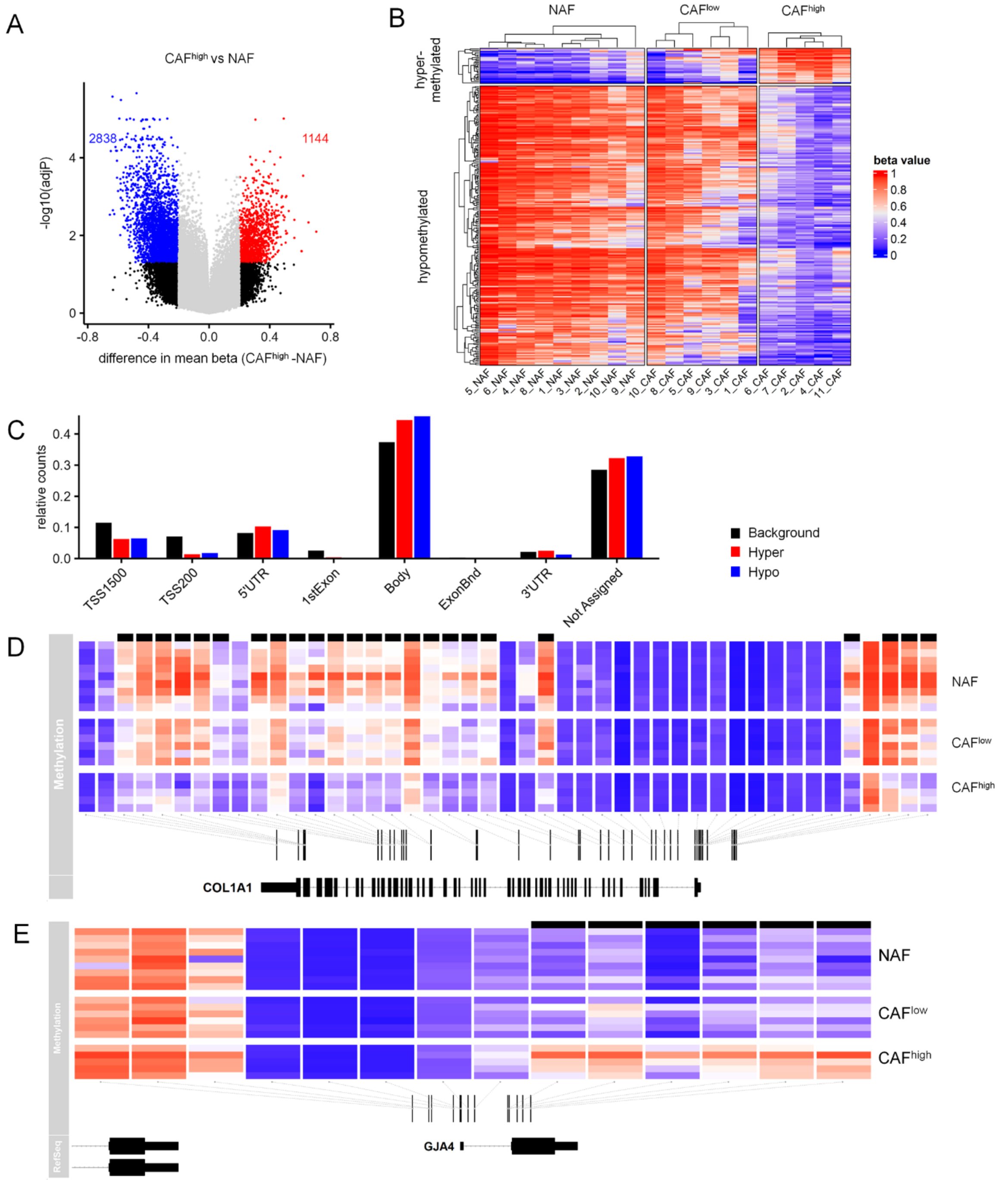

Figure S2: Differential DNAm between CAF^{high} and NAFs from liver tumors.

(A) Volcano plot comparing methylation data (beta values) from NAF and CAF^{high}. Adjusted p-values from limma are depicted.

(B) Heatmap of the top 250 differently methylated CpG sites with the highest difference in mean beta values between CAF^{high} and NAFs (limma adjusted p-values < 0.05).

(C) Distribution of differently methylated CpG sites to gene regions based on Illumina's annotation. The number of hyper- and hypo-methylated CpGs in the gene body was higher than the relative frequency of all CpGs on the array. In contrast, DNAm changes in promotor regions (TSS1500 and TSS200) were clearly underrepresented.

(D) Heatmap of DNAm of CpG sites associated with *COL1A1*. Black bars on top of the heatmap indicate significantly differently methylated sites between CAF^{high} and NAFs. *COL1A1* contained the top differently hypomethylated region (DMR).

(E) The top differently hypermethylated region was found overlapping the gene *GJA4*.

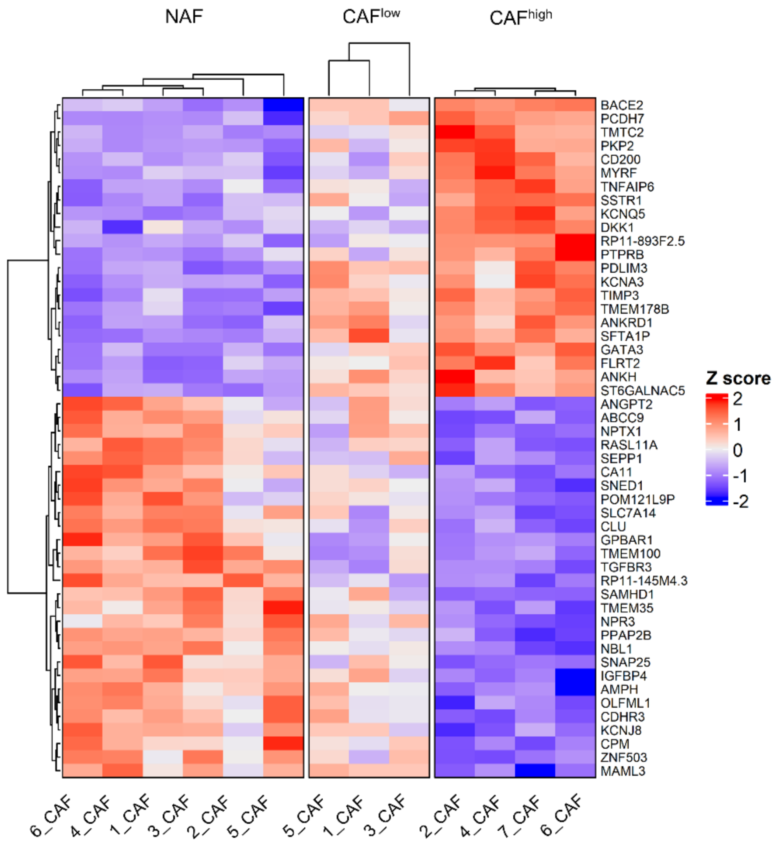

Figure S3: Differentially expressed genes in liver CAFs.

Top 50 most significantly different expressed genes between NAF and CAF^{high}. Depicted are Z scores of vst-transformed data from DEseq.

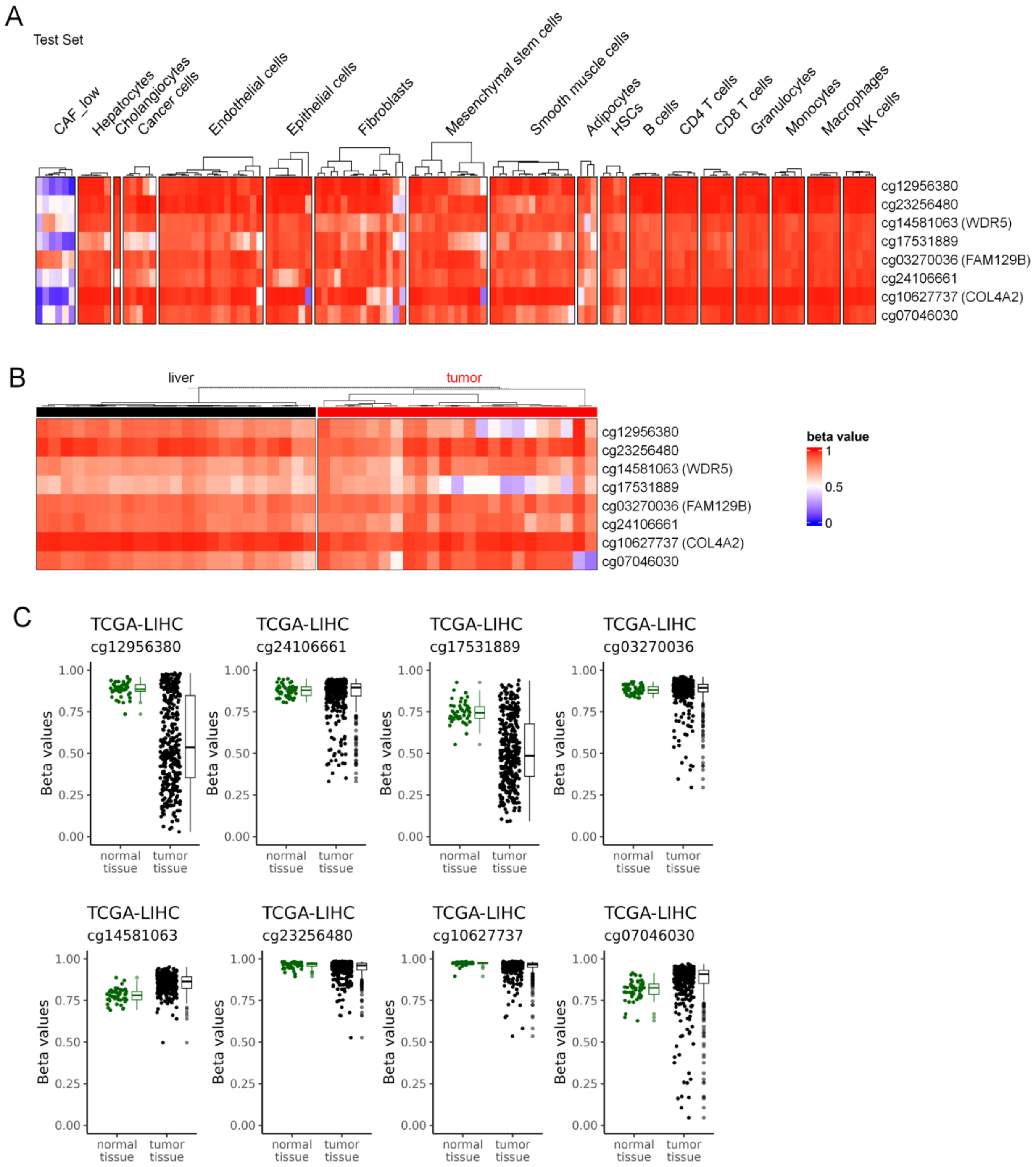

Figure S4: Further benchmarking of DNA methylation biomarkers for CAFs in liver cancer.

(A) Heatmap of DNAm of eight candidate CpGs that were selected to discern CAFs from other cell types. The results of the test dataset are depicted here.

(B) Heatmap of DNAm of the eight candidate CpGs for CAFs in normal liver tissue as compared to hepatocellular cancer (GSE136380).

(C) Furthermore, the eight candidate CpGs were analyzed in DNAm profiles of liver hepatocellular carcinoma (LIHC) of The Cancer Genome Atlas (TCGA). Box plots demonstrate differences between normal (green) and cancer tissue (black). Overall, cancer samples revealed higher variability than control samples.

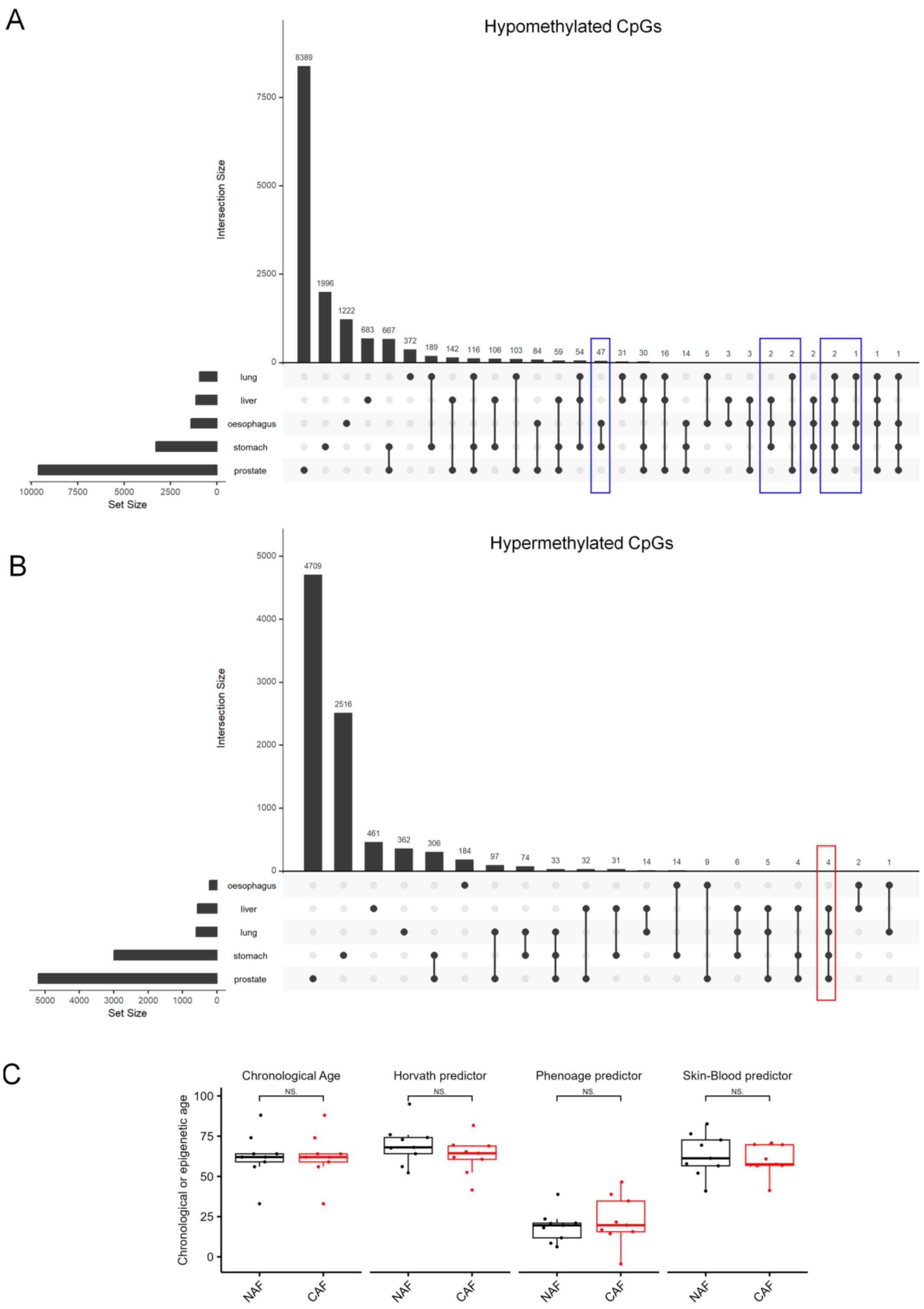

Figure S5: Overlapping CAF-associated CpGs in different datasets.

(A,B) Upset plots of the overlap of differently methylated CpG sites between each of the different tissues and cancers. Differential methylation analysis was performed for each dataset independently. Columns that show the overlap of differently methylated sites for at least four groups are marked. This analysis was performed for hypo- and hypermethylated CpGs.

(C) Age distribution and epigenetic age-predictions with three different clocks (Horvath, 2013; Horvath et al., 2018; Levine et al., 2018) in CAFs and NAFs. There was no clear difference in epigenetic age-predictions between CAFs and NAFs.

Supplemental Tables

Supplemental Table S1: Antibodies used in the study.

Antibodies	Label	Isotype	Clone	Manufacturer
Anti-human vimentin	-	Mouse IgM	LN-6, monoclonal	Sigma-Aldrich
Anti-human α-SMA	-	Mouse IgG2a	1A4, monoclonal	Sigma-Aldrich
Anti-human pan-cytokeratin	-	Mouse IgG1/ IgG2a	C-11+PCK-26+CY-90+KS-1A3+M20+A53-B/A2, monoclonal	Sigma-Aldrich
Anti-mouse IgM Alexa 594	-	Goat IgG	polyclonal	ThermoFisher
Anti-mouse IgG Alexa 647	-	Goat IgG	polyclonal	ThermoFisher
Anti-human CD14	APC	Mouse IgG2a	M5E2, monoclonal	BD Biosciences
Anti-human CD29	PE	Mouse IgG1	MAR4, monoclonal	BD Biosciences
Anti-human CD31	PE	Mouse IgG1	WM59, monoclonal	BD Biosciences
Anti-human CD34	APC	Mouse IgG1	581, monoclonal	BD Biosciences
Anti-human CD45	APC	Mouse IgG1	HI30, monoclonal	BD Biosciences
Anti-human CD73	PE	Mouse IgG1	AD2, monoclonal	BD Biosciences
Anti-human CD90	APC	Mouse IgG1	5E10, monoclonal	BD Biosciences
Anti-human CD105	FITC	Mouse IgG2a	MEM-226, monoclonal	ImmunoTools

Supplemental Table S4. Hazard ratios of COX regression models.

Cancer	CpG model	term	ci.95_lower	HR	ci.95_uper	adj_pval
KIRC	cg09809672	gendermale	0.72	1.08	1.62	0.92646
		age	1.016	1.034	1.052	0.00258
		cg09809672	0.003	0.012	0.049	< 0.00001
	cg07134930	gendermale	0.741	1.11	1.665	0.85663
		age	1.022	1.041	1.06	0.00021
		cg07134930	0	0.004	0.16	0.0294
	cg05935904	gendermale	0.691	1.039	1.56	0.94981
		age	1.016	1.034	1.053	0.00237
		cg05935904	0.015	0.066	0.296	0.00414
KIRP	cg09809672	gendermale	0.269	0.528	1.037	0.24555
		age	0.977	1.004	1.033	0.93486
		cg09809672	0.006	0.079	1.033	0.21173
	cg07134930	gendermale	0.269	0.529	1.039	0.24555
		age	0.981	1.008	1.035	0.82838
		cg07134930	0	0	0.007	0.00045
	cg05935904	gendermale	0.256	0.503	0.987	0.19766
		age	0.98	1.007	1.035	0.85663
		cg05935904	0.016	0.125	0.953	0.19766
LGG	cg09809672	gendermale	0.714	1.021	1.461	0.95686
		age	1.036	1.052	1.067	< 0.00001
		cg09809672	0	0	0.013	0.00031
	cg07134930	gendermale	0.746	1.067	1.525	0.92646
		age	1.043	1.057	1.073	< 0.00001
		cg07134930	0	0.039	6.719	0.46762
	cg05935904	gendermale	0.775	1.111	1.591	0.82997
		age	1.046	1.061	1.076	< 0.00001
		cg05935904	0.002	0.007	0.031	< 0.00001
LIHC	cg09809672	gendermale	0.631	0.908	1.306	0.85089
		age	0.996	1.01	1.024	0.42417
		cg09809672	0.048	0.182	0.692	0.07419
	cg07134930	gendermale	0.603	0.868	1.25	0.75219
		age	0.997	1.011	1.025	0.37921
		cg07134930	0.001	0.019	0.331	0.04297
	cg05935904	gendermale	0.609	0.879	1.268	0.78258
		age	0.998	1.012	1.026	0.33902
		cg05935904	0.401	0.983	2.413	0.98172
UVM	cg09809672	gendermale	0.638	1.535	3.689	0.63071
		age	1.009	1.05	1.093	0.08677
		cg09809672	0	0	0.003	0.00351
	cg07134930	gendermale	0.592	1.408	3.348	0.74469
		age	0.997	1.034	1.072	0.25712
		cg07134930	0.001	0.011	0.127	0.00339
	cg05935904	gendermale	0.502	1.222	2.977	0.8984
		age	1.018	1.061	1.107	0.03979
		cg05935904	0	0.002	0.044	0.00085

Hazard ratios (HR), 95% confidence intervals, and adjusted p-values are provided for all three relevant CpGs in five tumor models: KIRC = kidney renal clear cell carcinoma, KIRP = kidney renal papillary cell carcinoma, LGG = low grade glioma, LIHC = liver hepatocellular carcinoma, UVM = uveal melanoma. COX regression models were calculated for each cancer type and each of the three CpGs. Significant association with DNAm is highlighted in red.
